# Novel Strategies for Lung Cancer Interventional Diagnostics

**DOI:** 10.3390/jcm13237207

**Published:** 2024-11-27

**Authors:** Robert Smyth, Ehab Billatos

**Affiliations:** 1 Department of Medicine, Section of Pulmonary, Critical Care and Occupational Medicine University of Iowa, Iowa City, IA 52242, USA; rsmyth@uiowa.edu; 2 Department of Medicine, Section of Pulmonary and Critical Care Medicine, Boston University, Boston, MA 02215, USA; 3 Department of Medicine, Section of Computational Biomedicine, Boston University, Boston, MA 02215, USA

**Keywords:** lung cancer, biopsy, electromagnetic navigation bronchoscopy (ENB), radial endobronchial ultrasound (EBUS), robotic bronchoscopy, peripheral lesions, tissue acquisition, diagnostic yield, CT-guided biopsy, advanced imaging techniques

## Abstract

Lung cancer is a major global health issue, with 2.21 million cases and 1.80 million deaths reported in 2020. It is the leading cause of cancer death worldwide. Most lung cancers have been linked to tobacco use, with changes in cigarette composition over the years contributing to shifts in cancer types and tumor locations within the lungs. Additionally, there is a growing incidence of lung cancer among never-smokers, particularly in East Asia, which is expected to increase the global burden of the disease. The classification of non-small cell lung cancer (NSCLC) into distinct subtypes is crucial for treatment efficacy and patient safety, especially as different subtypes respond differently to chemotherapy. For instance, certain chemotherapeutic agents are more effective for adenocarcinoma than for squamous carcinoma, which has led to the exclusion of squamous carcinoma from treatments like Bevacizumab due to safety concerns. This necessitates accurate histological diagnosis, which requires sufficient tissue samples from biopsies. However, acquiring adequate tissue is challenging due to the complex nature of lung tumors, patient comorbidities, and potential complications from biopsy procedures, such as bleeding, pneumothorax, and the purported risk of local recurrence. The need for improved diagnostic techniques has led to the development of advanced technologies like electromagnetic navigation bronchoscopy (ENB), radial endobronchial ultrasound (rEBUS), and robotic bronchoscopy. ENB and rEBUS have enhanced the accuracy and safety of lung biopsies, particularly for peripheral lesions, but both have limitations, such as the dependency on the presence of a bronchus sign. Robotic bronchoscopy, which builds on ENB, offers greater maneuverability and stability, improving diagnostic yields. Additionally, new imaging adjuncts, such as Cone Beam Computed Tomography (CBCT) and augmented fluoroscopy, further enhance the precision of these procedures by providing real-time, high-resolution imaging. These advancements are crucial as lung cancer is increasingly being detected at earlier stages due to screening programs, which require minimally invasive, accurate diagnostic methods to improve patient outcomes. This review aims to provide a comprehensive overview of the current challenges in lung cancer diagnostics and the innovative technological advancements in this rapidly evolving field, which represents an increasingly exciting career path for aspiring pulmonologists.

## 1. Introduction

Lung cancer is the second most common solid organ malignancy worldwide and the most common among men. According to the International Agency for Research on Cancer (IARC) GLOBOCAN cancer statistics for the year 2020, there were an estimated 2.21 million cases of lung cancer (95% UI: 2.18–2.24) worldwide, representing the commonest cause of cancer death with 1.80 million deaths (95% UI: 1.77–1.82) [1]. This represents approximately one in 10 (11.4%) cancers diagnosed and one in five (18.0%) deaths from cancer. Most of the lung cancer we have seen over the last number of decades is a direct consequence of tobacco consumption patterns over the past century [2]. Changes in cigarette design and composition have led to a shift in prevalence of the location and histological subtype of lung cancer. With the increasing manufacture of filtered, low nicotine-and-tar-containing cigarettes, this has presumably led to smokers inhaling deeper to meet their nicotine requirements resulting in a more peripheral distribution of tobacco smoke in the lung [2,3]. The decrease in lung squamous cell carcinoma (SCC) is attributed to the reduction in polycyclic aromatic hydrocarbons in cigarette smoke, and the increase in tobacco-specific N-nitrosamines has shifted histology to adenocarcinoma [3]. This histological and locational change of cancer within the lung is one of the many problems now facing pulmonologists in patients with suspicious lung lesions on imaging.

Another complicating factor in identifying patients with lung cancer is the growing incidence in never-smokers [4], an evolving phenomenon that is attracting ongoing study to determine its origins [5]. Approximately one-third of all patients with lung cancer in East Asia are individuals who never smoked [6]. The incidence of lung cancer in Asia is anticipated to be a major contributor to the global lung cancer burden now and in the future [6].

Historically, the classification of NSCLC into more distinct subtypes was necessary due to advances in treatment and their differential response to chemotherapy in advanced disease. For example, Bevacizumab (Avastin) is a recombinant humanized monoclonal antibody that blocks angiogenesis by inhibiting vascular endothelial growth factor (VEGF) receptors. In 2004 a randomized phase II study of Carboplatin, Taxol, and Avastin showed a rate of a life threatening or fatal hemoptysis of 9%, disproportionately affecting the squamous carcinoma histological subtype [7]. Furthermore, Pemetrexed, a third generation, multi-targeted anti-folate cytotoxic that inhibits enzymes including thymidylate synthase, was found to be far more efficacious in patients with adenocarcinoma than squamous carcinoma [8]. These findings led the Food and Drug Administration (FDA) to exclude squamous carcinoma histology from treatment with these chemotherapeutic agents. Therefore, the endeavor to identify a precise histological sub-type of NSCLC became important not only in terms of treatment efficacy but also in terms of patient safety. This required increased tissue to allow for immunohistochemical staining for accurate diagnosis. Many diagnostic samples are cytologic, obtained from fine needle aspiration (FNA) via transthoracic needle aspiration or endobronchial ultrasound transbronchial needle aspiration (EBUS-TBNA) [9]. They lack tumor architecture and have limited cellular content [10]. Even the most accessible tumors that are accessible through the endobronchial route average only 20–25% of tumor tissue, and up to half of fragments reveal no tumor cells whatsoever [11]. One technique that preserves tissue morphology is cryobiopsy. A cryoprobe using carbon or nitrous oxide typically achieve freezing temperatures of −80 °C allowing the removal of otherwise friable tissue [12]. In a large prospective study of 593 patients with findings concerning lung cancer; the addition of cryobiopsy vs. flexible bronchoscopy alone improved diagnostic yields (95.2% vs. 82.2%, *p* = 0.0001) [13].

## 2. Tissue Acquisition Considerations

With the advent of neo-adjuvant therapies, the need for sufficient tissue for genomic and PDL-1 testing has increased enormously [14,15]. In the lung, this task can be difficult for a number of reasons.

(1) As the majority of patients are smokers, concurrent respiratory compromise is common [16], making diagnostic procedures difficult or impractical. The need for conscious sedation or general anesthesia during bronchoscopy can lead to respiratory depression that, in this patient population, may cause clinically significant desaturation [17], contributing to not only patient morbidity but also potentially a premature termination of the procedure.
(2) Diagnostic biopsies can cause bleeding (1.9%) as tumor tissue can be friable and highly vascular due to disordered angiogenesis. This is problematic within the lung as it is usually not compressible in the airway [18]. Airway bleeding can also result in hypoxia that may be life threatening. Similarly, bleeding into the pleural space post-biopsy can be difficult to detect clinically until at an advanced state, which subjects the patient to further invasive procedures. Indeed, a study investigating barriers to re-biopsy for the molecular classification of lung cancer found that in 18% of cases, it was mostly due to the patient being on anti-coagulation that the physician assessment concluded that the procedure was too high risk [19].
(3) The other major complication of lung biopsy is pneumothorax. This may require the need for further intervention with the placement of an intercostal tube into the thoracic cavity, invariably an in-patient admission and subsequent patient morbidity. Pneumothorax is most common in procedures where the lung tumor is accessed transthoracically with a CT-guided biopsy. Large case series have shown that patient features that increase this risk are smaller lesions or more peripheral lesions [20]. A recent meta-analysis of 46 eligible studies of transthoracic CT-core vs. CT-FNA showed a rate of pneumothorax of 25.3% and 18.8%, respectively, with 5.6% vs. 4.3% requiring further intervention. Major complication rates were 5.7% and 4.4%, respectively, overall [21]. Furthermore, recent evidence has shown that this technique may lead to local recurrence in the pleura of younger patients (<55 years old) with stage I disease [22].

Clearly, the current methods for the acquisition of lung cancer tissue by biopsy pose particular difficulties, not only due to the inherent characteristics of the tumors themselves but due to the patient characteristics associated with an older patient population with other medical co-morbidities. Clearly, new technologies are needed to improve tumor tissue yields.

Complicating this need for more tissue is the fortunate and encouraging shift towards earlier diagnosis with smaller lung nodules. The widespread introduction of lung cancer screening using low-dose CT scans of the chest has led to a stage shift towards earlier stage disease with 53.5% diagnosed with stage 1 disease in the largest examination of lung cancer screening in the U.S. to date [23]. Furthermore, the use of cross-sectional imaging has increased rapidly in North America [24], leading to a huge increase in incidentally found lung nodules in at-risk populations with an estimated 1.6 million nodules detected annually in the US even before the introduction of widespread screening [25]. As we detect lung cancer at an earlier stage in more peripheral parts of the lung, guidelines have recommended the least invasive method possible for these suspicious nodules [26]. The practice of interventional pulmonology and advanced bronchoscopy has expanded rapidly in recent years to rise to the occasion of accessing these small peripheral lesions in large part due to unprecedented technological advances. This has led to new approaches in diagnosing lung cancer at earlier stages while maximizing tissue acquisition for diagnostics. Lung cancer survival improves drastically when diagnosed at its earliest stage. As we move to screening at-risk groups with low-dose CT scans, smaller, peripheral lesions at earlier stages of disease will be detected (stage shift) [27]. Accessing these is usually only feasible with CT-guided methods, as conventional bronchoscopy has poor sensitivity [28], and the complication rate of CT-guided approaches remains high. Safer diagnostic techniques have been developed to address this problem.

## 3. Radial EBUS (rEBUS)

Radial endobronchial ultrasound (EBUS) has become a pivotal tool in the diagnosis of peripheral lung lesions, especially when traditional bronchoscopy is inadequate. Radial EBUS employs a radial mini-probe, which provides a circumferential, or 360-degree, ultrasound image of the lung tissue surrounding the probe. This imaging technique is highly valuable for visualizing peripheral pulmonary lesions that are beyond the reach of conventional bronchoscopes [29]. The radial mini-probe is inserted through the working channel of a flexible bronchoscope and advanced into the lung periphery. Once positioned, the probe emits ultrasound waves, creating a concentric ultrasound image that helps clinicians identify and characterize lesions. The radial EBUS system, unlike its linear counterpart used for mediastinal lymph node sampling, does not provide real-time guidance during biopsy but instead assists in locating the lesion and confirming its characteristics before the biopsy [30].

Again, diagnostic yields with this approach are higher when the probe can be passed within the lesion rather than adjacent to it. With a concentric ultrasound image, diagnostic accuracy (84%) [31] is similar to that of CT-guided biopsy. When the lesion is eccentric, this limitation can result in a lower diagnostic yield, especially when the lesion is not directly accessible or when there is an absence of a bronchus sign. The bronchus sign is often used to predict the success of both ENB and radial EBUS in achieving a diagnostic biopsy [32]. With regard to the safety profile, radial EBUS is generally considered safer than CT-guided biopsies due to its minimally invasive nature and lower complication rates. While CT-guided biopsies carry a significant risk of pneumothorax, radial EBUS’s approach reduces this risk. However, the lack of real-time guidance during biopsy can sometimes lead to sampling errors, particularly in complex cases where lesion localization is difficult.

## 4. Electromagnetic Navigation Bronchoscopy (ENB)

Electromagnetic navigation bronchoscopy (ENB) was developed in order to overcome the shortcomings of conventional bronchoscopy in the periphery of the lung. This system uses an electromagnetic field to precisely determine the exact location of the non-visible lesion on a standard endoscopic image.

Conventional bronchoscopy, while effective for central lesions, often falls short in diagnosing peripheral lung abnormalities due to the limited reach and visualization capacity of traditional bronchoscopes. One step forward to this was the development of virtual bronchoscopic navigation (VBN). A high-resolution CT allowed the construction of a virtual bronchial tree. This is displayed on a monitor next to the patient and real-time bronchoscopic images. It relies on a thin or ultra-thin bronchoscope to advance into the peripheral airways limiting its utility. Nevertheless, a prospective multicenter study comparing the addition of VBN to radial EBUS in 199 patients with small peripheral nodules showed a higher diagnostic yield in the VBN group with shorter procedure time [33]. ENB was developed to build on this approach by utilizing advanced technology to enhance the precision and effectiveness of bronchoscopic procedures. This technology integrates electromagnetic field technology with a standard bronchoscopic platform. The system creates a virtual, three-dimensional (3D) map of the patient’s airways using pre-procedural CT scans [34]. During the procedure, a sensor probe is navigated through the bronchial tree, guided by real-time electromagnetic tracking. This enables the clinician to precisely localize and biopsy lesions that are otherwise invisible in standard endoscopic images, an improvement on the VBN approach. The electromagnetic field allows the system to detect the position and orientation of the sensor probe within the lungs, providing unparalleled accuracy in targeting peripheral lesions [35].

One of the key factors influencing the diagnostic yield of ENB is the presence of a “bronchus sign” on pre-procedural imaging. The bronchus sign indicates that a bronchus leads directly to the lesion, which significantly enhances the likelihood of successful navigation and biopsy. The diagnostic yield of ENB can be high when the bronchus sign is present; however, in its absence, the success rate may decrease. Studies have shown that the overall diagnostic yield of ENB ranges from 60% to 85%, with the presence of the bronchus sign being a critical determinant of success [36,37,38]. The full results of the multi-center, randomized VERITAS trial comparing the diagnostic accuracy of CT-guided TTNA to ENB are eagerly awaited [39]. ENB is generally considered safer than CT-guided transthoracic needle biopsies, particularly concerning the risk of pneumothorax. Pneumothorax, a common complication of CT-guided biopsies, occurs less frequently with ENB, contributing to its favorable safety profile. Additionally, the minimally invasive nature of ENB, with fewer complications such as bleeding and infection, makes it a preferred option for patients with challenging lesions. Despite its benefits, ENB is associated with higher costs and longer procedure times compared to conventional bronchoscopy. The need for pre-procedural CT scans, specialized equipment, personnel expertise, and extended procedure times all contribute to the increased costs. Furthermore, the learning curve associated with ENB may initially prolong procedures, although this tends to decrease with experience [40].

## 5. Robotic Bronchoscopy

Ultrathin bronchoscopy and navigational bronchoscopy have advantages over transthoracic needle biopsies in terms of patient safety with lower rates of pneumothorax [37,39] and allow concomitant staging of the mediastinum with EBUS. More recently, results from neo-adjuvant immunotherapy trials have shown significant increases in event-free survival, making accurate tissue diagnosis and staging even more important [14,15]. Furthermore, there are concerns that transthoracic needle biopsies may lead to local recurrence in the pleura [22]. Robotic bronchoscopy has built on previous platforms like electromagnetic navigational bronchoscopy to provide a virtual pathway that allows for enhanced maneuverability and a further reach to safely navigate within the lung to biopsy lung tissue. However, robotic bronchoscopy provides a more stable platform than ENB that suffered from significant heterogeneity in diagnostic yields in real-world settings [41], while navigating further into the periphery than the ultrathin bronchoscopy approach [42].

**Figure 1:**
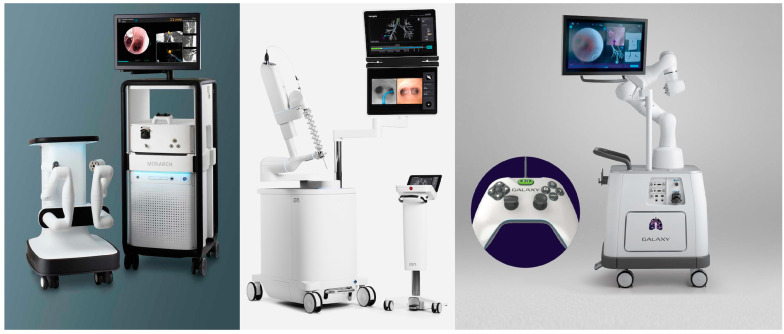
The Monarch, Ion, and Galaxy systems represent advancements in robotic bronchoscopy for the lung biopsy. Monarch (Ethicon Health) uses an electromagnetic field to guide two independently controlled arms, allowing precise movement, with a diagnostic yield of 74–77%. The Ion system (Intuitive Surgical) offers a thinner bronchoscope without a catheter, using shape-sensing technology for navigation and achieving a diagnostic yield of 81%. The Galaxy system (Noah Medical) is a disposable device using electromagnetic technology and digital synthesis to create a 3D image for guidance, with a low rate of pneumothorax and favorable safety profile.

The first robotic bronchoscopy platform was approved by the FDA in 2018 building on the now widely accepted use of robotic-assisted surgeries that are known to reduce tremor, allow 3D visualization, and have improved ergonomics for the operator (Figure 1). The first robotic bronchoscope approved was the Monarch^TM^ platform (Ethicon, CA, USA). Similar to the previous “SuperD” (SuperDimension^TM^, Medtronic, Minneapolis, MN, USA) system, it uses an electromagnetic field applied to the patient during the procedure after a dedicated chest CT has been performed. It has two independently functioning arms for the 6 mm catheter and the 4.2 mm bronchoscope. These are controlled by a console with two thumb controls that allow the operator to maneuver 130 degrees in any given direction. Although the definition of diagnostic yield varies by study, this platform reported a diagnostic yield of between 74% and 77%, using strict definitions with a sensitivity for malignancy of 82%. Successful lesion localization was seen in 96.2% of patients [43]. The Ion^TM^ system (Intuitive Surgical, Sunnyvale, CA, USA) better known for its well-established da Vinci surgical system is different to Monarch in many respects. Firstly, it consists of a single thinner bronchoscope 3.5 mm without a catheter and therefore does not allow for direct visualization during biopsy. Furthermore, it uses proprietary shape-sensing technology to localize and drive scope position within the lung rather than the electromagnetic field. The scope can articulate 180 degrees in a multi-directional fashion, and the scope is controlled by two roller balls. One purported benefit is a thinner scope compared to Monarch and the lack of electromagnetic fields that can cause interference. A diagnostic yield of 81% has been reported in two trials using this platform with a sensitivity for malignancy of between 87% and 79%, respectively [44,45]. A purportedly less costly option recently received regulatory approval. The Galaxy System^TM^ (Noah Medical, San Carlos, CA, USA) is a disposable bronchoscope that leverages electromagnetic technology in conjunction with proprietary digital synthesis technology—a technique where a series of X-ray images are captured at multiple angles to construct a 3-dimensional picture. These platforms have shown a favorable safety profile with low rates of pneumothorax between 0% and 5.8% [46].

Whatever platform is used, there are limitations to this technology that require the use of supplemental advanced imaging. The main reason for this is CT-to-body divergence. These procedures are more time consuming than standard flexible bronchoscopy and require the use of general anesthesia over conscious sedation. Furthermore, paralytics may also be used. 

It was increasingly appreciated that intra-procedure atelectasis was affecting the ability of these platforms to create an accurate pathway to the target lesion. Prior to the use of robotic bronchoscopy, a thin-slice chest CT is performed in a single breath hold at total lung capacity to allow the software to create a navigable pathway through the airways to the lesion of interest. However, during a prolonged procedure with the patient lying in recumbent position on a ventilator and often breathing near functional residual capacity, atelectasis was increasingly appreciated, particularly in the lower lobes [47]. This may lead to misleading feedback and a misregistration of the target lesion. Currently, intra-procedure ventilation strategies are being trialed to redress this issue [48].

## 6. Imaging Adjuncts

Unlike standard fluoroscopic images, which produce 2-dimensional (2D) visuals using a C-arm, advanced imaging techniques have significantly enhanced spatial resolution and accuracy, particularly in the context of robotic bronchoscopy. Among these advancements, Cone Beam Computed Tomography (CBCT) represents a significant leap forward in medical imaging. CBCT systems are typically mounted on the ceiling or floor of the procedure room, where a flat-panel detector captures cone-shaped X-rays. These X-rays are then used to reconstruct a 3D image of the chest, providing detailed anatomical information [49]. This method contrasts sharply with traditional CT imaging, which involves a larger radiation dose and less detailed spatial resolution. CBCT has been shown to deliver a lower effective dose of radiation while maintaining high diagnostic accuracy [50,51]. Studies have demonstrated that CBCT provides a diagnostic yield of 83.7% (95% CI: 74.8–89.9%) when used in conjunction with electromagnetic navigation bronchoscopy (ENB) [52].

The O-arm CT, also known as intraoperative CBCT, represents a varied iteration of CBCT technology. It utilizes a C-arm gantry that rotates around the patient, capturing real-time volumetric data through a cone-shaped X-ray beam and a flat-panel detector. This continuous rotation allows for the acquisition of high-resolution images that are reconstructed in real time, offering immediate feedback to the proceduralist. The O-arm CT system enhances the precision of navigation during bronchoscopy by providing updated 3D imaging that aids in accurate localization and biopsy of lesions [53].

Both CBCT and O-arm CT address key limitations of traditional imaging methods by improving spatial awareness and reducing radiation exposure. They are particularly valuable in procedures requiring detailed anatomical mapping and real-time feedback, thereby enhancing the effectiveness and safety of advanced bronchoscopy techniques. Augmented fluoroscopy incorporates real-time radiographic imaging with augmented reality overlay. This integrated system allows for real-time updates of the target lesion from pre-procedural imaging. Platforms such as LungVision^TM^ (Body Vision Medical Ltd., Weston, MA, USA) and CIOS Spin (Siemens, Forchheim, Germany) have been trialed to improve intra-procedure lesion localization and reduced CT-to-body divergence [54]. 

## 7. Full-Field Optical Coherence Tomography with Dynamic Cell Imaging (FFOCT-DCI)

While advanced bronchoscopy can improve our diagnostic yield, there is still uncertainty at the time of biopsy regarding the tumor cellularity of specimens or their adequacy for advanced testing for driver mutations or PD-L1. To address these challenges, researchers have developed several advanced techniques, including time-resolved optical imaging, frequency-domain optical imaging, confocal microscopy, and optical coherence tomography (OCT). Among these, OCT stands out as the preferred method for high-resolution optical imaging. OCT operates on the principle of white-light interference microscopy. To generate the interference signal, a reference mirror is oscillated using a piezoelectric transducer. The system captures en-face tomographic images by combining multiple images recorded by a high-speed CCD (charge-coupled device) camera at 200 Hz and enables three-dimensional reconstructions of internal structures. Full-field optical coherence tomography with dynamic cell imaging (FFOCT-DCI) is an advanced imaging technique that builds upon this technology using an incoherent light source and camera that offers high spatial and temporal resolution without sacrificing imaging depth. This technique captures entire en-face planes and provides sub-micrometer resolution by measuring temporal fluctuations of back-scattered light, allowing the visualization of sub-cellular structures and fast cellular dynamics. These combined technologies capture metabolic activity of a biopsy sample at a cellular level, enabling the proceduralist to perform interpretations in similar fashion to cytologic rapid on-site evaluation (ROSE).

**Figure 2:**
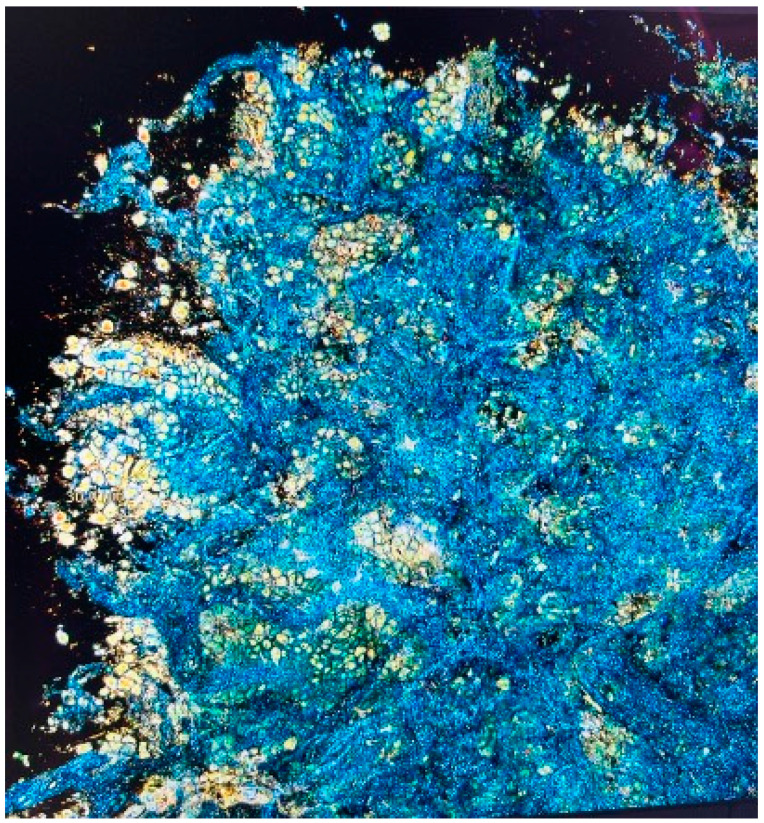
Full-field optical coherence tomography with dynamic cell imaging (FFOCT-DCI) is an advanced technique that provides high-resolution, three-dimensional imaging of biopsy samples using an incoherent light source. It captures sub-cellular structures and cellular dynamics, allowing real-time analysis similar to cytologic rapid on-site evaluation (ROSE). This method improves biopsy assessment by enabling proceduralists to evaluate cellularity and sample adequacy for advanced testing, enhancing diagnostic precision.

One study employing this novel biopsy assessment system (CelTivity, AquyreBiosciences™, Weston, MA, USA) enrolled 54 patients, from whom 43 peripheral lung lesions were sampled via robotic-assisted navigational bronchoscopy. The imaging technology performed well with a sensitivity of 90% and specificity of 77% relative to ROSE (Figure 2). Furthermore, the average time spent preparing the image was 1 min and 46 s suggesting its ability to be used in real-time diagnostic procedures (manuscript under review).

## 8. Liquid Biopsies

Although non-invasive liquid biopsies are by their nature not interventional or at best minimally interventional diagnostics, they are worth mentioning as an adjunct to invasive tissue sampling. Analysis of the components of the peripheral blood has been used for over a decade in the detection and management of non-small cell lung cancer. The use of cell-free DNA to detect targetable mutations has been incorporated into clinical guidelines because of their accuracy [55]. Their role in the diagnosis of early-stage lung cancer is less clear but appears to be nearing the point of clinical utility. Early CDT^®®^ Lung (OncImmune LLC, Cambridge, MA, USA) aims to detect the presence of tumor-associated antibodies in the blood of patients with concerning lung nodules. The diagnostic accuracy of this test was reported in a systematic review of 695 patients with a sensitivity of approximately 20.2% and specificity of 92% [56]. Gaga et al. validated the performance of Lung EpiCheck^®®^, a blood-based diagnostic that detects cancer-associated hypermethylation markers in cell-free DNA. This case-control study achieved AUCs of 0.882 and 0.899 in European and Chinese cohorts, respectively [57]. Another group has taken the novel approach of measuring the variance in the length of cell-free DNA strands to develop a diagnostic index to detect lung cancer in early-stage disease. Combining these fragmentation patterns with other clinical risk characteristics, they detected early-stage lung cancer (Stage I-II) successfully in 91% of patients [58] with further validation underway.

## 9. Conclusions

Newer bronchoscopy platforms and tissue imaging systems are still in the early phase of clinical implementation, and cost utility analyses are awaited pending larger scale real-world studies, but a recent health economic analysis using a decision analytic model suggests a potential economic benefit to these technologies [59]. These expensive and technically challenging platforms may be best used in high-volume centers with multi-specialty teams. Many smaller hospitals may struggle to justify the increased upfront costs of these tools or care for adequate numbers of patients to attain and then maintain procedural competency. Therefore, referral to high-volume centers seems a prudent approach for patients with solitary pulmonary nodules. Lung cancer therapeutic innovations have revolutionized treatments and improved overall survival. Technological advances in lung cancer diagnostics are catching up quickly. Studies to identify the right combination of novel tools are underway, and the rapidly evolving field represents an increasingly exciting career path for the aspiring pulmonologist.
